# Emergence of spatiotemporal invariance in large neuronal ensembles in rat barrel cortex

**DOI:** 10.3389/fncir.2015.00034

**Published:** 2015-07-08

**Authors:** Nathan S. Jacobs, Cynthia H. Chen-Bee, Ron D. Frostig

**Affiliations:** ^1^Department of Neurobiology and Behavior, University of California, IrvineIrvine, CA, USA; ^2^Department of Neurobiology and Behavior, Center for the Neurobiology of Learning and Memory, University of California, IrvineIrvine, CA, USA; ^3^Department of Biomedical Engineering, University of California, IrvineIrvine, CA, USA

**Keywords:** barrel cortex, stimulus invariance, multi-site recording, whisker array, sensory funneling

## Abstract

Invariant sensory coding is the robust coding of some sensory information (e.g., stimulus type) despite major changes in other sensory parameters (e.g., stimulus strength). The contribution of large populations of neurons (ensembles) to invariant sensory coding is not well understood, but could offer distinct advantages over invariance in single cell receptive fields. To test invariant sensory coding in neuronal ensembles evoked by single whisker stimulation as early as primary sensory cortex, we recorded detailed spatiotemporal movies of evoked ensemble activity through the depth of rat barrel cortex using microelectrode arrays. We found that an emergent property of whisker evoked ensemble activity, its spatiotemporal profile, was notably invariant across major changes in stimulus amplitude (up to >200-fold). Such ensemble-based invariance was found for single whisker stimulation as well as for the integrated profile of activity evoked by the more naturalistic stimulation of the entire whisker array. Further, the integrated profile of whisker array evoked ensemble activity and its invariance to stimulus amplitude shares striking similarities to “funneled” tactile perception in humans. We therefore suggest that ensemble-based invariance could provide a robust neurobiological substrate for invariant sensory coding and integration at an early stage of cortical sensory processing already in primary sensory cortex.

## Introduction

Invariance (also known as constancy, tolerance, or robustness) of sensory systems to major changes in sensory parameters is pivotal for survivability in a continuously changing sensory environment. How invariant sensory coding emerges at the neuronal level remains elusive. Neuronal invariance is typically studied in individual cortical neurons (Lueschow et al., 1994; Anderson et al., 2000; MacEvoy and Paradiso, 2001; Quiroga et al., 2005; Li and DiCarlo, 2008; Sadagopan and Wang, 2008). Coordinated activity of neuronal ensembles (Nicolelis and Lebedev, 2009; Quiroga and Panzeri, 2009; Buzsáki, 2010), could offer distinct advantages for invariant coding. For example, neuronal ensembles could mitigate notoriously variable responses in individual cortical neurons (Shadlen and Newsome, 1998). Invariance at the neuronal ensemble level could also rely on emergent response properties, such as spatiotemporal profiles of activity, which would be particularly relevant in topographically organized primary sensory cortices.

**Figure 1:**
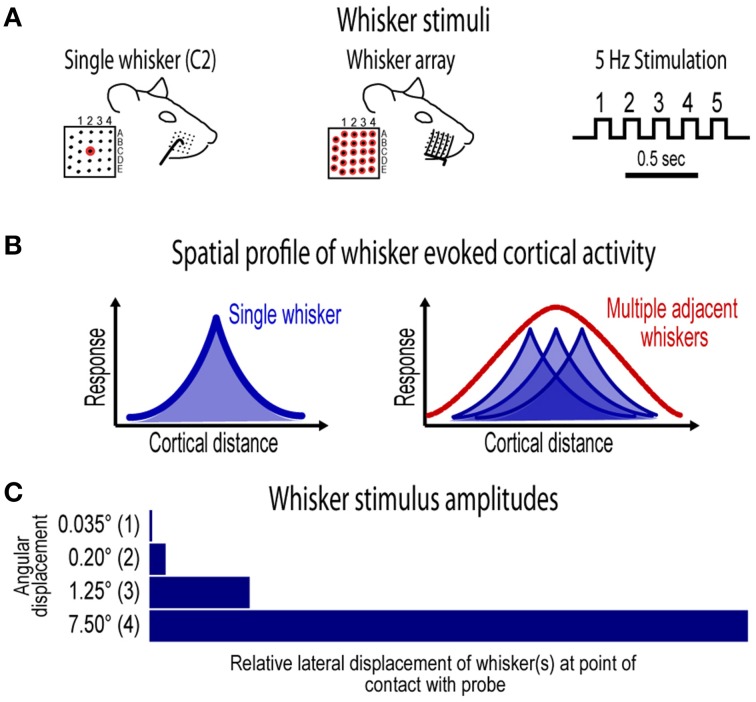
**Whisker stimuli and spatial profile of whisker evoked ensemble activity in barrel cortex**. **(A)** Ensemble-based invariance was investigated for two types of whisker stimuli- a single central whisker located at the center of the whisker pad (whisker “C2,” left) and the whisker array including all 24 large mistacial whiskers (middle). Whisker stimuli were delivered at a 5 Hz rate and consisted of 5 deflections per trial (right). **(B)** Schematics of previously reported spatial profiles of single whisker and whisker array evoked activity in barrel cortex (Chen-Bee et al., 2012). Note the large, overlapping profiles for single whisker and the single, central peak in the profile for whisker array. **(C)** Ensemble-based invariance was tested across logarithmic (base 6) changes in whisker stimulus amplitude that ranged from a barely visible movement of the whisker(s) at 0.035° to the relatively large stimulus amplitude of 7.5°.

**Figure 2:**
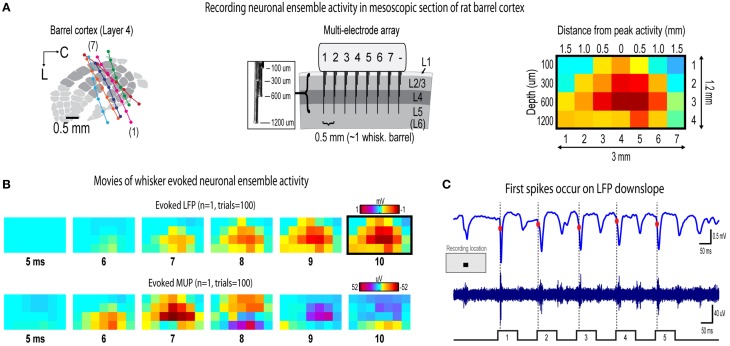
**Recording movies of continuous ensemble activity in barrel cortex**. **(A)** High fidelity 7 × 4 electrode arrays were used to acquire “snapshots” of ongoing activity across a mesoscopic section of cortex extending beyond the boundaries of rat barrel cortex and penetrating through most cortical layers (left and middle). **(B)** Representative movies of evoked local field potentials (LFP, top) and multi-unit potentials (MUP, bottom). The last frame of evoked LFP with dark border is same as in **(A)**. **(C)** It was initially surprising to see very early, small amplitude MUP signals occurring *before* LFP in **(B)**. However, when LFP (top) and MUP (bottom) filtered traces were compared during individual trials, the peak negative deflection of the first detected spike waveform occurred *during* LFP downslopes. The apparent discrepancy is resolved by noting the very early, small amplitude signals in trial averaged MUP (see “onset responses” in **Figures 4D, 6D**) that begin *before* the peak negative deflection of a single spike which are much more visible in individual traces.

Here we analyzed invariance of neuronal ensemble activity and its spatiotemporal characteristics in barrel cortex, a subdivision of primary somatosensory cortex in rodents. Observed from a mesoscopic vantage point, ensemble activity in barrel cortex is highly spatially organized. Single whisker evoke large “point spreads” of (mostly subthreshold) activity peaking over the appropriate barrel (Frostig et al., 2008), and following simultaneous multi-whisker stimulation unique, single peak integrated spatial patterns of activity emerge resulting from sublinear summation of simultaneously evoked point spreads (Chen-Bee et al., 2012 see schematics in Figure 1C). The aim of the current study was to assess the potential for spatiotemporal invariance of such neuronal ensembles following single whisker and whisker array stimulation by testing their potential for invariance to major changes in the amplitude of whisker stimuli (up to >200-fold changes; Figure 1C), as rats use and are sensitive to a wide range of whisker deflection amplitudes (Carvell and Simons, 1990) including very small amplitudes on the order of tens of microns (Simons, 1978; Jadhav et al., 2009). Movies of whisker evoked neuronal ensemble activity across a mesoscopic section of barrel cortex including most cortical layers were created from simultaneous multi-site recordings (Figure 2). Spatiotemporal profiles of evoked activity were then continuously monitored and quantified with <1 ms temporal resolution, revealing a remarkable degree of ensemble-based spatiotemporal invariance for both single whisker (whisker C2) and whisker array that includes all 24 large whiskers (vibrissae) evoked activity across the major changes in stimulus amplitude. These findings demonstrate invariant, spatially organized ensemble coding for both simple “point” stimuli (i.e., single whisker) as well as for more complex stimuli (i.e., whisker array) that involve integrated patterns of activity. Finally, we show how these findings could serve as the underlying neuronal correlate of simultaneous multi-site tactile perception in humans known as “funneling,” which is also amplitude-invariant (Békésy, 1967).

## Materials and methods

### Subjects and surgical preparation

Seven adult male Sprague–Dawley rats 2–3 months old were used in the study. All procedures were in compliance with the National Institutes of Health guidelines and reviewed and approved by the University of California Irvine Animal Care and Use Committee. Rats were inducted with sodium pentobarbital (55 mg/kg b.w.) and maintained with supplemental injections. Fast intrinsic signal optical imaging (Chen-Bee et al., 2010) of the C2 whisker barrel through an 8 × 8 mm region of thinned skull guided placement of electrodes. A small section of thinned skull and dura mater centered over the C2 whisker barrel was removed before insertion of electrode array. Complete insertion of electrodes was verified visually and with online monitoring of LFP traces. Cytochrome-oxidase staining of post-mortem tissue was used to verify location of electrode bundles within barrel cortex (Figure 2A).

### Whisker stimuli

Single whisker (whisker C2; Figure 1A, left) and whisker array (24 whiskers in rows A–E and arcs 1–4 plus all four Greek whiskers; Figure 1A, middle) stimulation was restricted to the right snout. Whiskers were deflected by 0.035°, 0.2°, 1.25°, or 7.5° using a single (for single whisker) or multiple (for whisker array) copper probe(s) mounted to a single arm controlled by a programmable stepping motor (Applied Motion Products, Watsonville, CA) and Master8 pulse generator (AMPI, Jerusalem, Israel). For each trial five whisker deflections were delivered at 5 Hz. For each condition, 100 trials were collected at 5 s intervals. Stimulus conditions were delivered pseudo-randomly such that all single whisker or whisker array conditions were completed before switching whisker probes. All subjects received all 8 whisker stimulus conditions (2 whisker stimulus types × 4 stimulus amplitudes) except one subject that only received whisker array conditions due to surgical complications which terminated the experiment early.

### Electrophysiology

Multi-site, extracellular recordings were acquired using 32-channel arrays with an 8 × 4 design consisting of 8 recording locations each of which had four depths targeting layers 1, 2/3, 4, and 5 (Figure 2A, middle). Electrode arrays were made from insulated 35 um tungsten wire (California Fine Wire, Grover Beach, CA; insulated with HML and VG bond coating) that were blunt cut and threaded in groups of four through polyimide guide tubes spaced 0.5 mm apart. Mean impedance of electrodes was 153 kΩ ± 55 (measured with IMP-2, Bak electronics, Sanford, FL). Raw signals starting 1 s before and ending 1 s after stimulus onset (total of 3 s per trial) were amplified and digitized at a 22 kHz sample rate (SnR system, Alpha Omega, Nazareth, Israel).

Analyses were done using custom MATLAB scripts. Raw traces were band-pass filtered for local field potentials (LFP, 1–300 Hz) or multi-unit potentials (MUP, 300–3k Hz) using a two-pole Butterworth function. LFP and MUP were averaged across trials. Trial averages of non-rectified, spike filtered traces have previously been interpreted as population firing synchrony (Temereanca and Simons, 2003). Trials with electrical noise (5.32% of trials) were excluded from trial averages. For the few bad channels in arrays (5.36% of channels overall, equivalent to 1.6 channels per array), trial averages from adjacent channels at the same cortical depth were averaged. In trial averages, mean baseline values 50 to 0 ms before stimulus onset were subtracted. A Gaussian filter was used to remove electrical noise near 60 Hz. For group analyses, trial averaged data was down-sampled to a 10 kHz sample rate. To spatially align data for group analyses, single whisker and whisker array data sets for each subject were shifted horizontally to align peak LFP responses in layers 2/3 and 4 for the strongest stimulus amplitude (7.5°). For suprathreshold responses, an abundance of overlapping spike waveforms (Supplementary Figure 1; see also Bar-Gad et al., 2001; Temereanca et al., 2008) made it difficult to interpret PSTHs of spike times although these results were still consistent with main findings (Supplementary Figures 2, 3).

### Spatiotemporal analyses

Electrophysiology data was analyzed at the mesoscopic level (i.e., data from all electrodes were analyzed concurrently). Frames of activity were normalized by dividing by the maximum value across all recording locations within 50 ms of stimulus onset (data for each deflection was normalized separately). Onset frames of activity were the first frames with a maximum value greater than the 99% confidence interval for pre-stimulus data 10 to 0 ms before stimulus onset. Peak frames of activity were frames with the maximum value within 50 ms of stimulus onset.

Principal component analysis (PCA) was performed on normalized data. Each frame of activity for each subject and condition (stimulus amplitudes 1–4) was vectorized and treated as a single observation without centering about the mean. Loadings for each principal component corresponded to how similar each frame of activity was to that particular principal component. Note that unlike correlations such loadings are sensitive to absolute magnitude, for example the weaker magnitudes in a frame of activity before peak responses would result in a reduced loading even if it had an identical relative profile of activity.

**Figure 3:**
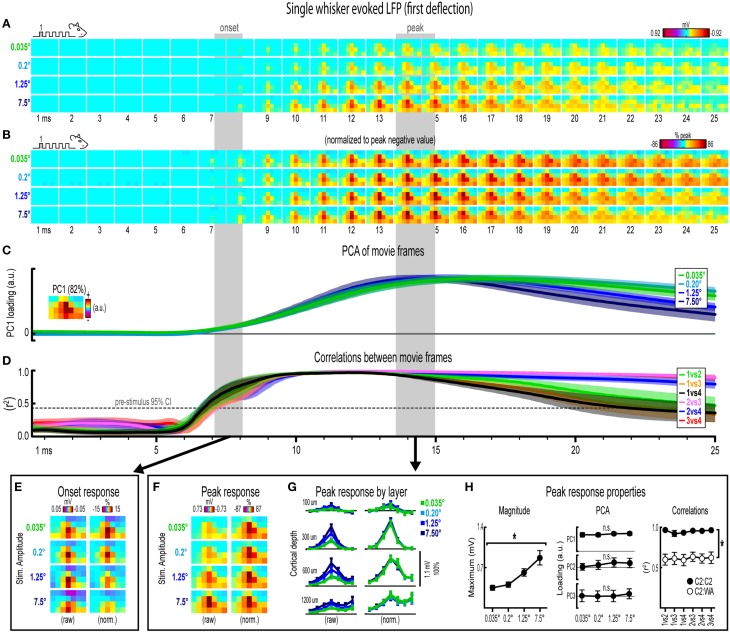
**Single whisker evoked LFP for the first deflection**. **(A)** Movies of averaged (*n* = 6) single whisker evoked LFP for the first deflection at each of the four stimulus amplitudes (0.035°, 0.2°, 1.25°, and 7.5°). Note the laminar and lateral spread of evoked LFP. **(B)** The relative spatial profile of evoked LFP spread can be compared across stimulus amplitudes by normalizing each movie to the maximum value across all pixels and time points. **(C,D)** Continuous quantification of spatial profiles with PCA loadings **(C)** and similarity between spatial profiles with correlations **(D)**. Traces are mean ± s.e.m. Gray shaded regions indicate mean onset and peak latencies (± s.e.m.). **(E)** Raw and normalized mean onset frames. **(F–H)** Raw and normalized mean peak frames **(F)**, broken down by layer **(G)**, and quantification of peak response properties **(H)**. ^*^p < 0.05.

**Figure 4:**
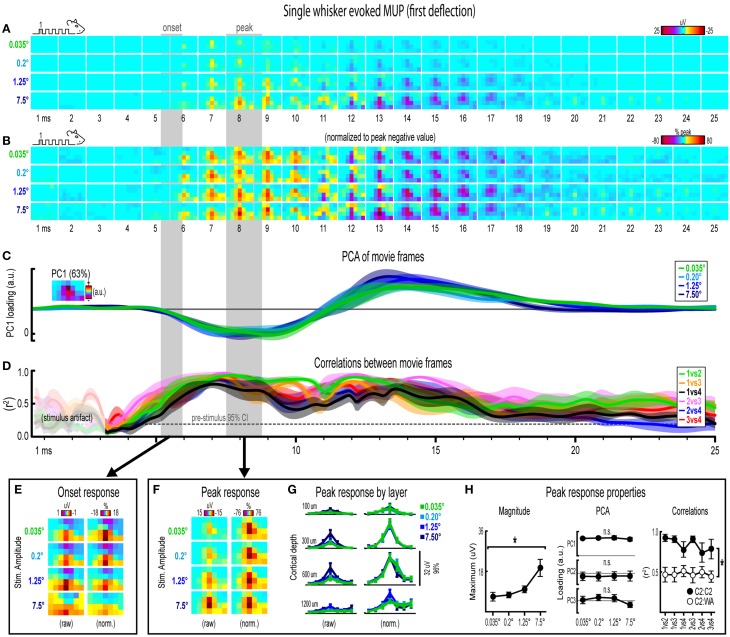
**Single whisker evoked MUP for the first deflection**. **(A,B)** Raw **(A)** and peak-normalized **(B)** movies of averaged (*n* = 6) single whisker evoked MUP for the first deflection.** (C,D)** Continuous quantification of spatial profiles with PCA loadings **(D)** and similarity between spatial profiles with correlations **(E)**. Traces are mean ± s.e.m. Gray shaded regions indicate mean onset and peak latencies (± s.e.m). **(E)** Raw and normalized mean onset frames. **(F–H)** Raw and normalized mean peak frames **(F)**, broken down by layer **(G)**, and quantification of peak response properties **(H)**. ^*^p < 0.05.

**Figure 5:**
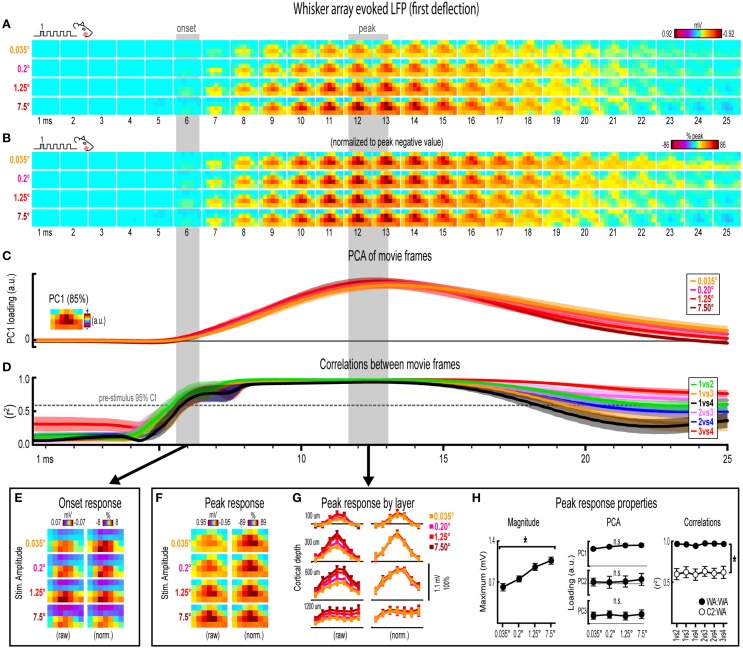
**Whisker array evoked LFP for the first deflection. (A,B)** Raw **(A)** and peak-normalized **(B)** movies of averaged (*n* = 7) whisker array evoked LFP for the first deflection.** (C,D)** Continuous quantification of spatial profiles with PCA loadings **(D)** and similarity between spatial profiles with correlations **(E)**. Traces are mean ± s.e.m. Gray shaded regions indicate mean onset and peak latencies (± s.e.m). **(E)** Raw and normalized mean onset frames. **(F–H)** Raw and normalized mean peak frames **(F)**, broken down by layer **(G)**, and quantification of peak response properties **(H)**. ^*^p < 0.05.

**Figure 6:**
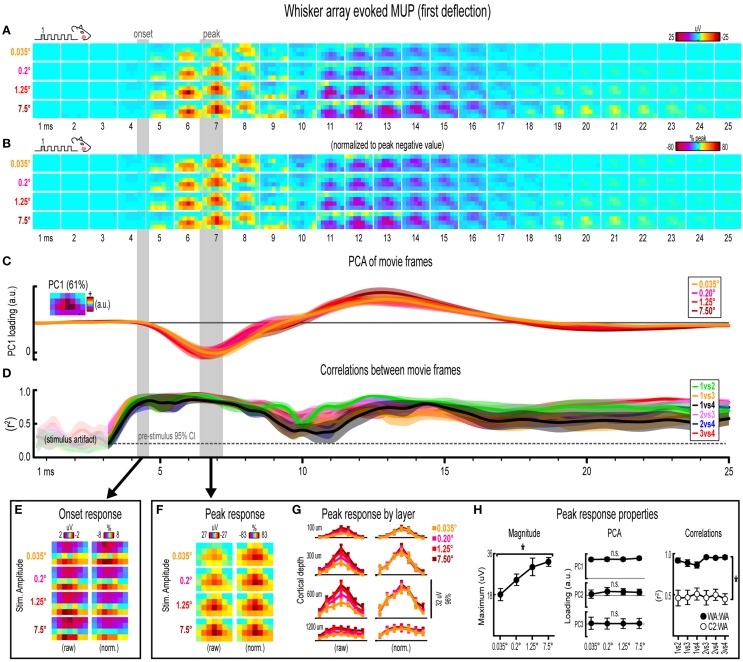
**Whisker array evoked MUP for the first deflection. (A,B)** Raw **(A)** and peak-normalized **(B)** movies of averaged (*n* = 7) whisker array evoked MUP for the first deflection.** (C,D)** Continuous quantification of spatial profiles with PCA loadings **(D)** and similarity between spatial profiles with correlations **(E)**. Traces are mean ± s.e.m. Gray shaded regions indicate mean onset and peak latencies (± s.e.m). **(E)** Raw and normalized mean onset frames. **(F–H)** Raw and normalized mean peak frames **(F)**, broken down by layer **(G)**, and quantification of peak response properties **(H)**. ^*^p < 0.05.

**Figure 7:**
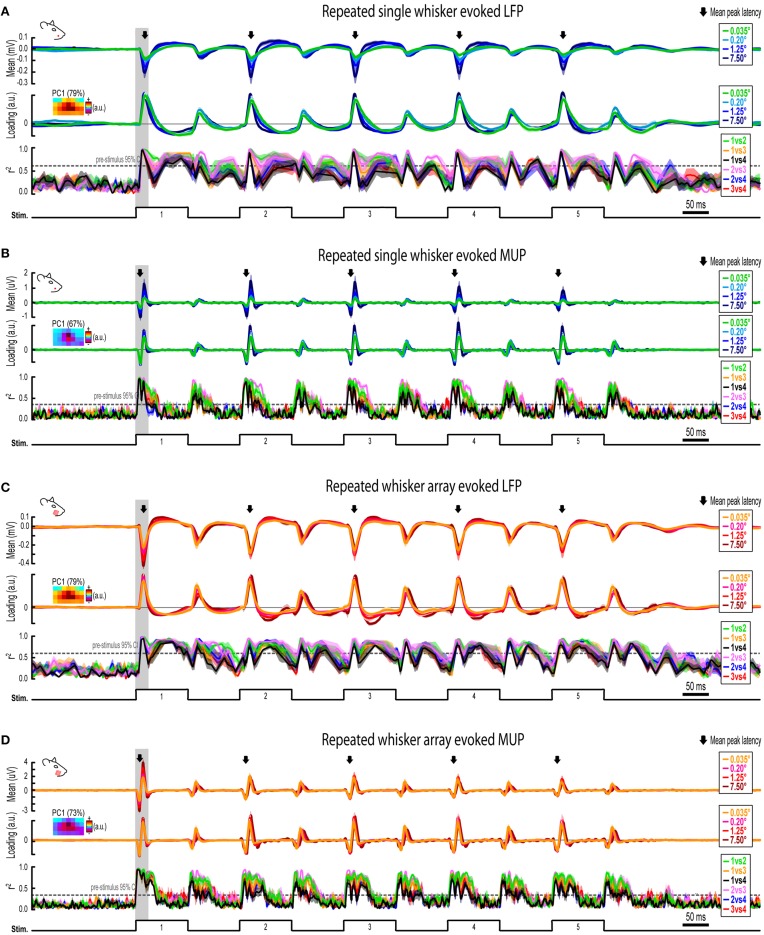
**Continuous quantification of evoked LFP and MUP for repeated whisker deflections. (A)** Continuous quantification of the magnitude (top panel), spatial profile (middle panel), and similarity of spatial profiles (bottom panel) for single whisker evoked LFP. **(B–D)** Same data as **(A)** but for single whisker evoked MUP **(B)**, whisker array evoked LFP **(C)**, and whisker array evoked MUP **(D)**. Note the much larger time window (~2 s) compared to previous figures. *Arrows indicate mean latencies of peak frames of evoked activity which are further analyzed in Figure 7. All traces indicate mean ± s.e.m*.

Pearson's correlations were calculated separately for each subject and group averages reported (mixed-model). Confidence intervals for pre-stimulus *r*^2^-values were calculated from all stimulus amplitude comparisons using a pre-stimulus time window (−10 to 0 ms for analysis of 25 ms window in Figures 3–6E and -200 to 0 ms for analysis of 1.4 s window in Figure 7). Quartile-quartile plots of data at pre-stimulus, onset, and peak responses revealed no major deviations from a normal distribution. All spatiotemporal analyses were done within subjects and group statistics reported.

### Statistical analyses

All parametric statistics (repeated measures ANOVA, paired *t*-tests) were performed in SYSTAT version 11. For grand means, multiple values for each subject were first averaged before grand mean and s.e.m. calculations.

## Results

**Figure 8:**
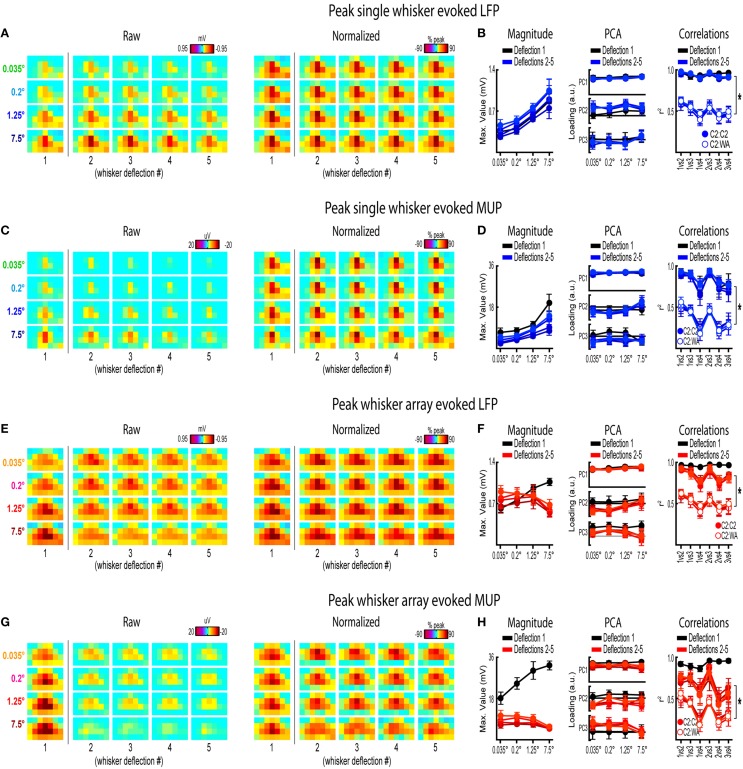
**Peak frames of activity for repeated whisker deflections**. Mean peak frames and quantification of peak response properties for single whisker evoked LFP **(A,B)**, single whisker evoked MUP **(C,D)**, whisker array evoked LFP **(E,F)**, and whisker array evoked MUP **(G,H)** for repeated whisker deflections. *Results from the first deflection are plotted for comparison*. ^*^p < 0.05.

The current project assessed invariance in spatiotemporal profiles of whisker evoked ensemble activity in rat barrel cortex across major changes in stimulus amplitude. Two types of whisker stimulation were used, single whisker (C2) and whisker array (all 24 large whiskers), and were delivered at a naturalistic 5 Hz rate for a total of five whisker deflections per trial (Figure 1A). For both whisker stimuli, ensemble activity was assessed across logarithmic (base 6) changes in whisker stimulus amplitude (Figure 1C). The smallest stimulus amplitude (0.035°) was barely perceptible to the eye and the largest stimulus amplitude (7.5°) was comparable to our previous studies (Frostig et al., 2008; Chen-Bee et al., 2012). Movies of whisker evoked activity were recorded across a mesoscopic section of cortex that extended through and beyond barrel cortex and penetrated through most cortical layers (Figure 2). The exact positioning of electrode arrays was constrained by blood vessel patterns in each subject producing some variability across subjects, however all spatiotemporal analyses were performed within subjects eliminating any between-subjects differences. For suprathreshold responses, trial averaged multi-unit potentials (referred to hereafter as “MUP”; see methods for details) were preferred over PSTHs because of an abundance of overlapping spike waveforms that made spike counts uninterpretable (Supplementary Figure 1). Spatial profiles of evoked activity were continuously monitored with high temporal resolution (< 1 ms) and compared across major changes in stimulus amplitude (up to >200-fold). Results for the first of five whisker deflections, analogs to a single deflection of a whisker or the whisker array, are presented in Figures 3–7. Results for repeated single whisker or whisker array deflections, analogs to repetitive whisking behaviors, are presented in Figures 7, 8.

### Invariance in single whisker evoked LFP for the first deflection

Movies of single whisker (C2) evoked local field potentials (LFP) for the first of five deflections and their quantification are shown in Figure 3. For the first deflection, movies are shown from 1 to 25 ms post-stimulus onset. The same analyses used in this section are repeated in following sections. As discussed in detail below, major changes in stimulus amplitude (up to > 200-fold) affected the magnitude but *not* the spatiotemporal profile of single whisker evoked MUP for the first deflection.

Mean single whisker evoked LFP (*n* = 6) for each of the four stimulus amplitudes (0.035°, 0.2°, 1.25°, 7.5°) is shown in Figure 3A. Single whisker evoked LFP for each stimulus amplitudes spread vertically and laterally across the field of view within the 25 ms time window. Despite differences in absolute magnitude, a similarly broad lateral profile of evoked LFP for each stimulus amplitude was observed by normalizing each movie to the peak negative value across all frames within the 25 ms window (Figure 3B). Further support for the large spatial profile of evoked LFP was that each stimulus amplitude engaged a similarly large region of cortex including all recording locations within the field of view, all of which had evoked LFP within the 25 ms window at least three standard deviations above pre-stimulus data (Supplementary Figure 4A). Thus, initial qualitative assessment revealed notably similar spatiotemporal profiles of single whisker evoked LFP for the first deflection.

Spatiotemporal profiles of single whisker evoked LFP for the first deflection were quantified and compared across stimulus amplitudes (Figures 3C,D). The spatial profile of evoked LFP at each time point was quantified using principle component analysis (PCA) of normalized data (Figure 3C). The first principal component (PC1; Figure 3C, top, inset) explained 82% of the variance in frames of evoked LFP, with PC2 and PC3 explaining only 6 and 4% of the variance, respectively. PC1 loadings were then plotted over time for each stimulus amplitude (Figure 3C, top). Note that identical spatiotemporal profiles would result in identical (i.e., completely overlapping) traces of PC1 loadings. Traces of PC1 loadings for each stimulus amplitude were highly overlapping from the onset of responses (left shaded region in Figure 3C) through peak responses (right shaded region in Figure 3C). PCA results therefore matched initial findings of similar spatiotemporal profiles of single whisker evoked LFP for the first deflection.

Similarity between frames of single whisker evoked LFP for the first deflection was quantified with correlations between all possible stimulus amplitude pairs (1 vs. 2, 1 vs. 3, 1 vs. 4, 2 vs. 3, 2 vs. 4, and 3 vs. 4) at each time point within the 25 ms window (Figure 3D). Note that the “1 vs. 4” comparison (black traces in Figure 3D) between the smallest (0.035°) and largest (7.5°) stimulus amplitude represented a ~215-fold difference in stimulus amplitude. At ~7 ms post-stimulus onset, mean coefficients of determination (*r*^2^-values) rose above a 95% confidence interval (gray dotted line in Figure 3D) calculated from pre-stimulus data for all stimulus amplitude comparisons. Mean *r*^2^-values for all comparisons appeared to reach a maximum at ~10 ms and then slowly tapered off after peak. These data further substantiated the similarity in spatiotemporal profiles of evoked LFP observed in peak-normalized movies (see Figure 3B). Together, continuous qualitative and quantitative measures suggested highly similar spatiotemporal profiles of single whisker evoked LFP were maintained despite major changes in stimulus amplitude.

Lastly, onset and peak frames of single whisker evoked LFP for the first deflection warranted closer inspection. Mean onset and peak latencies were 7.6 ± 0.4 ms and 14.2 ± 0.6 ms, respectively (shaded regions in Figures 3A–D; grand mean of subject and stimulus amplitude ± s.e.m.; see Supplementary Table 1 for details). Onset responses were difficult to see in Figure 3A due to their small magnitude. Therefore, onset frames of evoked LFP were aligned, averaged, and plotted with a “zoomed in” color scale that was ~ 20 times more sensitive (Figure 3E). The more sensitive color scale revealed a consistent pattern of positive or neutral voltages at the most superficial depth targeted at layer 1 (top row of pixels in each image) and negative voltages in the two deepest depths targeted at layers 4 and 5 (bottom two rows of pixels in each image) for all stimulus amplitudes. Similar patterns were found for all onset frames of evoked LFP and MUP (see relevant sections below), and were likely produced by fast, synchronous activity in thalamocortical afferents (Kandel and Buzsáki, 1997).

Peak frames of single whisker evoked LFP for the first deflection were aligned and averaged (Figure 3F). The same data was also broken down by recording depth to allow closer inspection of laminar responses (Figure 3G). Similar to before, response magnitudes increased with increasing stimulus amplitude but had nearly indistinguishable spatial profiles. Supporting the observed change in response magnitude, the maximum value in peak frames was significantly different across stimulus amplitudes [*F*_(3, 15)_ = 16.12, *p* < 0.001; Figure 3H, left]. Supporting the similarity of spatial profiles, PC1, PC2, and PC3 loadings for peak normalized frames were not significantly different across stimulus amplitudes [*PC1*, *F*_(3, 15)_ = 0.36, *p* = 0.780; *PC2*, *F*_(3, 15)_ = 2.44, *p* = 0.105; *PC3*, *F*_(3, 15)_ = 2.09, *p* = 0.145; Figure 3H, middle].

Further supporting the similarity of spatial profiles, peak frames were also highly correlated with each other (mean *r*^2^ = 0.95 ± < 0.01; grand mean of comparison and subject; Figure 3H, right, closed circles). A simple internal control was used to test the sensitivity of correlations by comparing single whisker to whisker array responses, which are both characterized by the same basic shape (a single, central peak of activity; see schematics in Figure 2B). Importantly, this control comparison resulted in significantly lower *r*^2^-values [Figure 3H, right, open circles; grand mean *r*^2^ = 0.62 ± 0.06; *F*_(1, 5)_ = 107.86, *p* < 0.001; for peak frames of whisker array evoked LFP see Figure 5F], indicating that correlations were highly sensitive to even subtle changes in the profile of evoked activity.

Results for single whisker evoked LFP for the first deflection suggested that large changes in stimulus amplitude (up to > 200-fold) affected the magnitude but not the spatiotemporal profile of activity.

### Invariance in single whisker evoked MUP for the first deflection

Movies of single whisker evoked multi-unit potentials (MUP) for the first of five deflections and their quantification are shown in Figure 4A. The exact same analyses used before were repeated and are summarized briefly below. Note that MUP responses had an early negative and a late positive peak within the 25 ms post-stimulus time window. All analyses of peak MUP responses focused on the earlier negative peak. Similar to before, major changes in stimulus amplitude (up to > 200-fold) again affected the magnitude but not the spatiotemporal profile of single whisker evoked MUP for the first deflection.

Mean single whisker evoked MUP (*n* = 6) for the first deflection increased in magnitude with increasing stimulus amplitude (Figure 4A), had notably similar spatiotemporal profiles as revealed by normalizing to peak values (Figure 4B), and included evoked activity >3 standard deviations above pre-stimulus data across the entire field of view (Supplementary Figure 4B). Continuous quantitative measures further supported the finding of similar spatiotemporal profiles across stimulus amplitudes. For PCA (Figure 4C), PC1 explained 63% of the variance with PC2 and PC3 explaining only 12% and 7%, respectively. Traces of mean PC1 loadings for each stimulus amplitude were again highly overlapping, even during transitions between negative and positive MUP phases. For correlations between frames of evoked MUP (Figure 4D), mean *r*^2^-values for all stimulus amplitude comparisons rose above the 95% pre-stimulus confidence interval at 4–5 ms post-stimulus onset, appeared to reach a maximum by 7 ms, and were highest during peak negative and peak positive responses. Together, continuous qualitative and quantitative measures suggested highly similar spatiotemporal profiles of single whisker evoked MUP were maintained despite major changes in stimulus amplitude.

Onset and peak frames of single whisker evoked MUP for the first deflection were again inspected more closely. Onset and peak latencies were 5.6 ± 0.4 ms and 8.1 ± 0.5 ms on average (shaded regions in Figure 4; see Supplementary Table 1 for details). Onset frames of evoked MUP (Figure 4E) again had positive or neutral voltages in the most superficial depth and negative voltages in the two deepest depths for each stimulus amplitude. Peak frames of evoked MUP (Figures 4F–H) again demonstrated changes in response magnitude but not spatial profile. Supporting the observed change in response magnitude, the maximum value within peak frames was significantly different across stimulus amplitudes [*F*_(3, 15)_ = 4.47, *p* = 0.02; Figure 4H, left]. Supporting the similarity of spatial profiles, PC1, PC2, and PC3 loadings for peak frames were not significantly different across stimulus amplitudes [*PC1*, *F*_(3, 15)_ = 0.33, *p* = 0.805; PC2, *F*_(3, 15)_ = 0.64, *p* = 0.602; *PC3*, *F*_(3, 15)_ = 2.01, *p* = 0.156; Figure 4H, middle]. Further supporting the similarity of spatial profiles, peak frames were also highly correlated with each other (Figure 4H, right, closed circles; grand mean *r*^2^ = 0.94 ± 0.01). Importantly, peak frames of single whisker evoked MUP were significantly less correlated with peak frames of *whisker array* evoked MUP [Figure 4H, right, open circles; grand mean *r*^2^ = 0.51 ± 0.06; *F*_(1, 5)_ = 12.09, *p* = 0.018; for peak frames of whisker array evoked MUP see Figure 6F), again indicating that correlations were sensitive to even subtle changes in profiles of activity.

Together, results for single whisker evoked MUP and LFP for the first deflection suggested that large changes in stimulus amplitude (up to > 200-fold) affected the magnitude but not the spatiotemporal profile of neuronal ensemble activity.

### Invariance in whisker array evoked LFP for the first deflection

Movies of whisker array evoked LFP for the first of five deflections and their quantification are shown in Figure 5. The exact same analyses used before were repeated and are summarized briefly below. Similar to before, major changes in stimulus amplitude (up to > 200-fold) again affected the magnitude but not the spatiotemporal profile of whisker array evoked LFP for the first deflection. Z-scores for whisker array evoked activity within the 25 ms window are shown in Supplementary Figures 4C,D.

Mean whisker array evoked LFP (*n* = 7) for the first deflection increased in magnitude with increasing stimulus amplitude (Figure 5A), had notably similar spatiotemporal profiles as revealed by normalizing to peak values (Figure 5B), and included evoked activity >3 standard deviations above pre-stimulus data across the entire field of view (Supplementary Figure 4E). Continuous quantitative measures further supported the finding of similar spatiotemporal profiles across stimulus amplitudes. For PCA (Figure 5C), PC1 explained 85% of the variance with PC2 and PC3 both explaining only ~4% of the variance. Traces of mean PC1 loadings for each stimulus amplitude were again highly overlapping. For correlations between frames of evoked LFP (Figure 5D), mean *r*^2^-values for all stimulus amplitude comparisons rose above the 95% confidence interval for pre-stimulus data at ~6 ms, reached a maximum by ~8 ms before slowly tapering off. Together, continuous qualitative and quantitative measures suggested highly similar spatiotemporal profiles of whisker array evoked LFP for the first deflection were maintained despite major changes in stimulus amplitude.

Onset and peak frames of whisker array evoked LFP for the first deflection were again inspected more closely. Onset and peak latencies were 6.0 ± 0.4 ms and 12.3 ± 0.6 ms on average (shaded regions in Figure 5; see Supplementary Table 1 for details). Onset frames of evoked LFP (Figure 5E) had positive or neutral voltages in the two most superficial depths and negative voltages in the two deepest depths for each stimulus amplitude. Peak frames of evoked LFP (Figures 5F–H) again demonstrated changes in response magnitude but not spatial profile. In Figure 5G, note the single, central peaks of activity at all recording depths except the deepest targeted at layer 5. Supporting the observed change in response magnitude, the maximum value within peak frames was significantly different across stimulus amplitudes [*F*_(3, 18)_ = 14.75, *p* < 0.001; Figure 5H, left]. Supporting the similarity of spatial profiles, PC1, PC2, and PC3 loadings for peak frames were not significantly different across stimulus amplitudes [*PC1*, *F*_(3, 18)_ = 2.80, *p* = 0.069; *PC2*, *F*_(3, 18)_ = 0.35, *p* = 0.793; *PC3*, *F*_(3, 18)_ = 0.63, *p* = 0.608; Figure 5H, middle]. Further supporting the similarity of spatial profiles, peak frames were also highly correlated with each other (Figure 5H, right, closed circles; grand mean *r*^2^ = 0.96 ± < 0.01). Importantly, peak frames of whisker array evoked LFP were significantly less correlated with peak frames of *single whisker* evoked LFP [Figure 5H, right, open circles; grand mean *r*^2^ = 0.62 ± 0.06; *F*_(1, 5)_ = 3447.61, *p* < 0.001; for peak frames of single whisker evoked LFP see Figure 3F], again indicating that correlations were sensitive to even subtle changes in profiles of activity.

Results for whisker array evoked LFP for the first deflection again suggested that major changes in stimulus amplitude (up to > 200-fold) affected the magnitude but not the spatiotemporal profile of neuronal ensemble activity, but this time for the more complex whisker array stimulation involving all 24 large whiskers evoking a distinct pattern of sensory integration.

### Invariance in whisker array evoked MUP for the first deflection

Movies of whisker array evoked MUP for the first of five deflections and their quantification are shown in Figure 6. The exact same analyses used before were repeated and are summarized briefly below. Note again the tendency of spatial profiles to be characterized by a single, central peak of activity at superficial recording depths targeted at layers 1, 2/3, and 4 but not the deepest targeted at layer 5. All analyses of peak MUP responses again focused on the earlier negative peak. Similar to before, major changes in stimulus amplitude (up to > 200-fold) again affected the magnitude but not the spatiotemporal profile of whisker array evoked MUP.

Mean whisker array evoked MUP (*n* = 7) for the first deflection increased in magnitude with increasing stimulus amplitude (Figure 6A), had notably similar spatiotemporal profiles as revealed by normalizing to peak values (Figure 6B), and included evoked activity >3 standard deviations above pre-stimulus data across the entire field of view (Supplementary Figure 4F). Continuous quantitative measures further supported the finding of similar spatiotemporal profiles across stimulus amplitudes. For PCA (Figure 6C), PC1 explained 61% of the variance with PC2 and PC3 explaining only 16 and 7%, respectively. Traces of mean PC1 loadings for each stimulus amplitude were again highly overlapping, even during transitions between negative and positive MUP phases. For correlations between frames of evoked MUP (Figure 6D), mean *r*^2^-values for all stimulus amplitude comparisons rose above the 95% pre-stimulus confidence interval just before onset latencies and were highest during peak negative and peak positive responses. Together, continuous qualitative and quantitative measures suggested highly similar spatiotemporal profiles of whisker array evoked MUP for the first deflection were maintained despite major changes in stimulus amplitude.

Onset and peak frames of whisker array evoked MUP for the first deflection were again inspected more closely. Onset and peak latencies were 4.4 ± 0.2 ms and 6.8 ± 0.4 ms on average (shaded regions in Figure 6; see Supplementary Table 1 for details). Onset frames of evoked MUP (Figure 6E) had strongly positive voltages in the two most superficial depths and neutral or negative voltages in the two deepest depths for each stimulus amplitude. Peak frames of evoked MUP (Figures 6F–H) again demonstrated changes in response magnitude but not spatial profile. In Figure 6G, again note the single, central peaks of activity at all recording depths except the deepest targeted at layer 5. Supporting the observed change in response magnitude, the maximum value within peak frames was significantly different across stimulus amplitudes [*F*_(3, 18)_ = 11.74, *p* < 0.001; Figure 6H, left]. Supporting the similarity of spatial profiles, PC1, PC2, and PC3 loadings were not significantly different across stimulus amplitudes [*PC1*, *F*_(3, 18)_ = 1.36, *p* = 0.287; *PC2*, *F*_(3, 18)_ = 0.68, *p* = 0.579; *PC3*, *F*_(3, 18)_ = 0.25, *p* = 0.859; Figure 6H, middle]. Further supporting the similarity of spatial profiles, peak frames of whisker array evoked MUP were also highly correlated with each other (Figure 6H, right, closed circles; grand mean *r*^2^ = 0.92 ± 0.02). Importantly, peak frames of whisker array evoked MUP were significantly less correlated with peak frames of *single whisker* evoked MUP [Figure 6H, right, open circles; grand mean *r*^2^ = 0.51 ± 0.06; *F*_(1, 5)_ = 65.84, *p* = 0.001; for peak frames of single whisker evoked MUP see Figure 4F), again indicating that correlations were sensitive to even subtle changes in profiles of activity.

Together, results for the first single whisker and whisker array deflection suggest that major changes in whisker stimulus amplitude (up to > 200-fold) had a significant effect on the magnitude but not spatiotemporal profile of evoked activity. This finding held for the relatively simple deflection of a single, central whisker (C2) and for the more complex whisker array stimulation involving simultaneous stimulation of all 24 large whiskers. The similarity in profiles of activity was supported by highly sensitive quantitative measures that despite failing to detect differences across stimulus amplitudes could detect differences between two similar whisker stimuli- namely single whisker and whisker array responses both characterized by a single, central peak of activity. Correlation results consistently passed this sensitivity test. PCA also seemed to pass this sensitivity test when performed on a combined data set including both single whisker and whisker array responses (Supplementary Figure 5). Overall, results for the first single whisker and whisker array deflection suggested that major changes in stimulus amplitude systematically affected the magnitude of evoked activity but did not produce any substantial changes in the profile of evoked activity.

### Invariance during repeated whisker deflections

Rodents explore their environment with repetitive, simultaneous movement of their whiskers. It was therefore important to determine if results from the first deflection, analogs to a single deflection of the whisker(s), extended to repeated whisker deflections 2–5. A similar set of analyses were performed on results during repeated whisker deflections with a few important differences. Now, a larger time window (−0.2–1.2 s post-stimulus onset) was used that included all five whisker deflections of the 5 Hz stimulation. For each movie, the mean magnitude within each frame of activity was calculated and continuously plotted (Figures 7A–D, top panels). Similar to before, mean PC1 loadings and mean *r*^2^-values for all possible stimulus amplitude comparisons were continuously plotted (Figures 7A–D, middle and bottom panels, respectively). The gray shaded regions in Figure 7 correspond to the 25 ms time window used for analysis of the first deflection (see Figures 3–6). Arrows in Figure 7 indicate time of peak responses (mean negative peak latency within 50 ms of stimulus onset for each deflection; see Supplementary Table 1 for details). All further analyses focused on peak frames of evoked activity (Figure 8).

Repeated single whisker deflections 2–5 continued to evoke LFP and MUP that increased in magnitude with increasing stimulus amplitude but did not have major changes in its spatiotemporal profile (Figures 8A–D). The maximum value within peak frames was significantly different across stimulus amplitudes [*LFP*, Figure 8B, left, *F*_(3, 15)_ = 8.43, *p* = 0.002; *MUP*, Figure 8D, left, *F*_(3, 15)_ = 8.29, *p* = 0.002]. The spatial profile of peak frames did not change noticeably across stimulus amplitudes (Figures 8A,C, right). For PCA results, PC1 explained a majority of variance (79% for LFP and 67% for MUP), with PC2 and PC3 again explaining much less of the variance (between 5 and 11%). No significant differences in PC1, PC2, or PC3 loadings for LFP or MUP data were found across stimulus amplitudes *except* for PC3 for LFP which explained only 5% of the variance [*F*_(3, 15)_ = 23.21, *p* < 0.001; Figure 8B, “PC3” in middle panel] and PC2 for MUP which explained only 11% of the variance [*F*_(3, 15)_ = 3.53, *p* = 0.041; Figure 8D, “PC2” in middle panel], and overall no major differences in spatial profiles were noticeable (see Figures 8A,C, right). Peak frames were well-correlated with each other (LFP, *r*^2^ = 0.94 ± 0.01; MUP, *r*^2^ = 0.82 ± 0.03; grand mean of comparisons, deflections, and subjects) and were significantly less correlated with peak frames of *whisker array* evoked activity [LFP, *r*^2^ = 0.51 ± 0.05, *F*_(1, 5)_ = 132.05, *p* < 0.001, Figure 8B, right; MUP, *r*^2^ = 0.36 ± 0.04, *F*_(1, 5)_ = 142.00, *p* < 0.001, Figure 8D, right]. These data suggest that stimulus amplitude continued to affect the magnitude but not the spatiotemporal profile of evoked LFP and MUP for repeated single whisker deflections.

In contrast to all previous results, repeated whisker array deflections evoked LFP and MUP that did not increase in magnitude despite major increases in stimulus amplitude (up to >200-fold; Figures 8E,G, left). There were still some significant differences in the maximum value within peak frames [*LFP*, Figure 8F, left, *F*_(3, 18)_ = 3.33, *p* < 0.043; *MUP*, Figure 8H, left, *F*_(3, 18)_ = 4.47, *p* = 0.016], however all *post-hoc* tests were not significant [*all F*_(1,6)_ *<* 15, *all p* > 0.008, *Bonferroni correction for 6 comparisons*]. If anything, the *largest* stimulus amplitude appeared to evoke the *weakest* response magnitudes (Figures 8F–H, left, red lines). Similar to previous results, the spatial profile of peak frames did not show any major changes across stimulus amplitudes (Figures 8E,G, right). For PCA results, PC1 again explained the majority of variance (79% for LFP and 73% for MUP) with PC2 and PC3 again explaining much less of the variance (between 4 and 8%). No significant differences in PC1, PC2, or PC3 loadings for LFP or MUP data were found across stimulus amplitudes *except* for PC2 for LFP which explained only 6% of variance [*F*_(3, 18)_ = 5.76, *p* = 0.006; Figure 8F, “PC2” in middle panel] and PC3 for MUP which explained only 6% of variance [*F*_(3, 18)_ = 5.28, *p* = 0.009; Figure 8H, “PC3” in middle panel], and overall no major differences in spatial profiles were noticeable (see Figures 8E,G, right). Peak frames were well-correlated with each other (LFP, *r*^2^ = 0.84 ± 0.04; MUP, *r*^2^ = 0.65 ± 0.05; grand mean of comparisons, deflections, and subjects) and were significantly less correlated with peak frames of *single whisker* evoked activity [LFP, *r*^2^ = 0.51 ± 0.05, *F*_(1, 5)_ = 60.07, *p* = 0.001, Figure 8F, right; MUP, *r*^2^ = 0.36 ± 0.04, *F*_(1, 5)_ = 46.98, *p* = 0.001, Figure 8H]. These data suggest that the spatiotemporal profile of whisker array evoked LFP and MUP continued to be relatively invariant to even major changes in stimulus amplitude during repeated deflections. Further, in contrast to all previous results, these data also suggest that the absolute magnitude of whisker array evoked LFP and MUP may also become invariant to stimulus amplitude for repeated deflections.

Results from repeated whisker deflections indicate that the spatiotemporal profile of neuronal ensemble activity in rat barrel cortex continued to be notably invariant to even major changes in stimulus amplitude (up to >200-fold). The absolute magnitude of responses, however, consistently increased with increasing stimulus amplitude except, notably, for the more naturalistic repeated deflections of the whisker array.

### Whisker array responses faster, less variable across subjects

Two main differences between single whisker and whisker array responses were observed. First, whisker array responses were faster than single whisker responses. LFP onset latencies were significantly faster for whisker array compared to single whisker conditions (*paired t-test of mean onset latencies for all stimulus amplitudes*, *t*_(5)_ = 5.76, *p* = 0.002; see Supplementary Table 1 for all latency values). LFP peak latencies were faster for whisker array compared to single whisker but not significantly so (*paired t-test of mean peak latencies for all stimulus amplitudes*, *t*_(5)_ = 2.37, *p* = 0.064). MUP onset and peak latencies were both significantly faster for whisker array compared to single whisker (*paired t-tests of mean onset and peak latencies; MUP onset latency*, *t*_(5)_ = 5.61, *p* = 0.003; *MUP peak latency*, *t*_(5)_ = 4.59, *p* = 0.006).

In general, the onset latencies for trial averaged MUP data were consistent but at the low end of previously reported latencies in barrel cortex using spike timestamps (e.g., Armstrong-James et al., 1992). The shorter latencies in trial averaged MUP could be explained by increased sensitivity to small amplitude signals which are necessarily excluded in thresholded data used for spike detection. Contributions from small amplitude signals could originate from: the rising phase of action potentials, action potentials from smaller cells such as spiny stellate cells, and synchronized activation of thalamocortical afferents (Kandel and Buzsáki, 1997; for detailed review of origins of extra-cellular currents see Buzsáki et al., 2012).

The second main difference between single whisker and whisker array responses was that maximum response magnitudes were less variable across subjects for the first whisker array deflection. The coefficient of variance (COV, standard deviation divided by mean) for whisker array evoked LFP for the first deflection was 0.19 (mean COV for all stimulus amplitudes), 34% lower than the COV for single whisker evoked LFP for the first deflection which was 0.29. The COV for whisker array evoked MUP for the first deflection was 0.28, 45% lower than the COV for single whisker evoked MUP for the first deflection which was 0.51. For repeated deflections, COVs were not consistently different between single whisker and whisker array conditions.

## Discussion

The current research investigated invariance in large, spatially organized neuronal ensembles of rat barrel cortex. Several methods used here (e.g., combined analysis of continuous multi-site recordings) enabled direct comparison of spatial profiles of evoked activity with high temporal resolution over relatively long periods of time. We found that neuronal ensemble activity has a remarkable capacity for spatiotemporal invariance. Such ensemble-based spatiotemporal invariance was found for a single whisker stimulus as well as for a more complex whisker array stimulus involving many whiskers and a distinct pattern of sensory integration.

### Emerging invariance in neuronal ensembles

Neuronal invariance is typically studied at the level of single neurons, which in “higher” sensory cortices can invariantly respond to abstract sensory information such as objects or items (Sáry et al., 1993; Lueschow et al., 1994; Li and DiCarlo, 2008; Rust and DiCarlo, 2010). In primary sensory cortices invariance has been observed in more nuanced aspects of individual neuron responses such as the width of tuning curves (Anderson et al., 2000; Sadagopan and Wang, 2008), yet very little is known about invariance at the neuronal ensemble level in primary sensory cortex.

Here we analyzed a special case of neuronal ensemble: the “point spread,” which describes the rapid lateral spread of evoked activity following point sensory stimulation (e.g., whisker). Point spreads are ubiquitous in sensory cortex (somatosensory, auditory, and visual) ranging from mice and rats to cats and monkeys and are found in both anesthetized and awake behaving animals (Grinvald et al., 1994; Barth et al., 1995; Das and Gilbert, 1995; Bakin et al., 1996; Bringuier et al., 1999; Brett-Green et al., 2001; Kaur et al., 2005; Ferezou et al., 2006, 2007; Roland et al., 2006; Sharon et al., 2007; Frostig et al., 2008; Chen-Bee et al., 2012; Mohajerani et al., 2013). Interestingly, multiple simultaneous point spreads propagating through presumably overlapping neuronal ensembles have been shown to summate (Chen-Bee et al., 2012; Gao et al., 2012). A potential criticism of studying point spreads in the anesthetized preparation is that anesthesia may result in unnaturally large point spreads. However, this does not seem to be the case as single whisker evoked point spreads in barrel cortex are equally as large or larger in awake vs. anesthetized rodents (Ferezou et al., 2006).

Point spreads in the rat barrel cortex are supported by a robust lateral connectivity based on long-range, roughly radially symmetric horizontal projections (e.g., Frostig et al., 2008; Mohajerani et al., 2013; Stehberg et al., 2014; Johnson and Frostig, 2015). Just like the gradually tapering activity profile evoked by single whisker stimulation, it is apparent that the density of such non-specific horizontal fibers also gradually tapers with cortical distance from an individual whisker barrel (see Johnson and Frostig, 2015). A recent study has directly linked large spatial profiles of cortical activity with underlying spatial patterns of lateral structural connectivity in mice (Mohajerani et al., 2013). Recruitment of a larger proportion of the cells and horizontal fibers of this lateral network could explain why stronger stimulus intensities increase response magnitudes in a spatially uniform manner, although this remains to be fully tested.

Why are point-spreads so ubiquitous, especially in light of the expensive metabolic support that cortex has to invest in order to maintain them? We have previously shown, using stimulus amplitude comparable to the largest stimulus amplitude in the current study, that single whiskers stimulation evokes point spreads that have a considerable degree of spatial overlap even for topographically distant whiskers (Chen-Bee et al., 2012). Importantly, summation of these overlapping point spread accurately predicts a single peak of evoked activity following simultaneous stimulation of all 24 large whiskers (Chen-Bee et al., 2012); and therefore point spreads could be described as a “building block” of integrated cortical activity. Here we expand the importance of point spreads by demonstrating their spatiotemporal invariance. Specifically, spatiotemporal profiles of single whisker evoked activity were notably invariant despite major changes in whisker stimulus amplitude that exceeded 200-fold differences. Further, we reasoned that if point-spreads are indeed building blocks of cortical integrated activity, then this spatiotemporal invariance should also extend to the patterns of multi-point integration they construct. Indeed, a similar degree of spatiotemporal invariance was also found for whisker array evoked neuronal ensemble activity across the same major changes in stimulus amplitude. These findings therefore seem to generalize the critical role of interactions among single whisker evoked point spreads across a wide range of ethologically relevant whisker stimulus amplitudes. Taken together, the building block function and its invariance suggest that point spreads should be considered as important players in cortical functional organization.

The ensemble-based invariance reported here also demonstrates how emergent properties of large neuronal ensembles (e.g., the relative profile of activity across constituent neurons in the ensemble) can be independent of absolute response magnitude. Sensory coding independent of response magnitude may allow simultaneous coding of stimulus intensity (e.g., stimulus amplitude) and other more nuanced stimulus features (e.g., texture). Such simultaneous sensory coding could help explain why stimulus intensity often does not affect recognition of specific objects or items.

We further suggest that in primary sensory cortices ensemble-based invariance may be more biologically relevant than invariance at the individual neuron level. Invariant response features do exist at the individual neuron level in primary sensory cortex (e.g., the tuning curve widths mentioned earlier), but require comparing responses occurring at different times and to different stimuli thus raising important questions about how exactly this information could be used in real time (Quiroga and Panzeri, 2009). In contrast, the ensemble-based invariance described here relies on emergent response features (e.g., the relative profile of activity) that can be used in real-time presumably by so called “reader” cells in downstream cortical areas (Buzsáki, 2010). Combined with the current findings, these observations strongly suggest that neuronal ensembles are not only capable of a remarkable degree of invariance but, given their emergent response properties which allow for continuous, magnitude-independent sensory coding, appear better designed to perform this function than individual neurons.

### Habituation during repeated whisker array deflections

Interestingly it seems that for the more naturalistic stimulation, repeated deflections of the entire whisker array, an additional level of neuronal invariance may occur in the absolute magnitude of responses. In a study of single unit responses in barrel cortex, it was reported that increasing the *frequency* of repeated whisker array deflections increases response magnitude (Mowery et al., 2011). Surprisingly, the current results suggest that this is not the case for stimulus amplitude. We found that repeated whisker array deflections (i.e., beyond the first stimulation) seemed to equilibrate absolute response magnitudes for each stimulus amplitude. The same equilibration of response magnitudes was not observed for repeated single whisker deflections, suggesting that the underlying mechanism may be specific to simultaneous stimulation of many whiskers. These findings, together with noticeable differences in response latencies between the first and repeated deflections (see Supplementary Table 1) and known adaptation of responses in the rodent somatosensory system (Chung et al., 2002; Katz et al., 2006; Temereanca et al., 2008), suggest distinct differences in sensory coding for repeated whisker array deflections.

### Relevance to funneled tactile perception

It has been previously established that the spread of subthreshold evoked activity in the anesthetized sensory cortex could serve as a correlate of perceptual phenomenon (Jancke et al., 2004). Could our findings also relate to tactile perception?

The single, central peak of evoked cortical activity observed after simultaneous stimulation of two or more adjacent points in the periphery has been suggested as the underlying neuronal correlate of “funneled” tactile perception (Chen et al., 2003; Chen-Bee et al., 2012), originally described by Békésy (1957, 1958, 1959, 1967). Békésy and colleagues demonstrated that multiple oscillating tactile stimuli applied simultaneously at several discrete skin sites are perceived as a *single* central stimulus, rather than as multiple points, leading him to describe the altered spatial profile of the perceived stimulus as being “funneled” into the central stimulus location.

The current findings show that a similarly “funneled” spatial profile of evoked activity in barrel cortex is invariant across a wide range of ethologically relevant whisker stimulus amplitudes, matching original observations that funneled tactile perception is amplitude-invariant (Békésy, 1959). These results further strengthen our previous suggestion that the integrated, spatial profile of evoked cortical activity following simultaneous multi-point stimulation could serve as the underlying neuronal correlate of funneled tactile perception. The current study replicates funneled profiles of cortical activity in superficial cortical layers (targeted at layers 1–4). However, funneled responses were not observed in deeper cortical layers (targeted at layer 5), possibly due to differences in the spatial organization of whisker evoked activity in infragranular layers of barrel cortex as compared to the other cortical layers (Armstrong-James et al., 1992; Sakata and Harris, 2009).

Similar to funneled tactile perception in humans which improves response latencies (Hashimoto et al., 1999), it is possible that simultaneous stimulation of multiple adjacent whiskers in the rat is perceived as a single highly responsive “super whisker” facilitating neuronal and behavioral responses that are faster, more reliable, and less variable. Consistent with this notion, improved tactile discrimination accuracy and faster behavioral response latencies have been associated with simultaneous multi-whisker stimulation in rodents (Celikel and Sakmann, 2007). Furthermore, decreased variability in neuronal responses in barrel cortex has also been associated with whisker array stimulation (Chen-Bee et al., 2012). Here we also report that neuronal responses in barrel cortex were also significantly faster for whisker array stimulation.

Summarizing the relationship to funneled tactile perception, evoked cortical activity in barrel cortex has a matching spatial profile, has similar latency and variability improvements compared to single point stimuli, and is also invariant to stimulus amplitude at the neuronal ensemble level. Together, these results provide a compelling case for the involvement of mesoscopic, spatially organized ensembles in the robust coding and integration of tactile information in somatosensory cortex.

It is possible that similar emergent patterns of ensemble activity have computational roles in other sensory regions such as primary visual and auditory cortices, which are also spatially organized. Differences in coding properties and network structure in these other sensory areas are likely to facilitate differing computational contributions. For example, spatial integration in primary visual cortex could result in a peak of activity between simultaneously activated orientation columns, rather than between whisker columns. However, general properties of ensemble-based sensory coding such as its invariance to absolute response magnitudes and high information content on single trials are likely relevant to all spatially organized ensembles in sensory cortex. Future research can now be pursued to determine whether the emergence of invariance within large, spatially organized neuronal ensembles can be generalized to other stimulus parameters and cortical areas.

## Author contributions

NJ and RF conceived and designed experiments. NJ performed all experiments and designed and performed all data analyses. NJ and RF wrote manuscript. CB assisted with data analysis and provided comments and feedback on manuscript.

### Conflict of interest statement

The authors declare that the research was conducted in the absence of any commercial or financial relationships that could be construed as a potential conflict of interest.
