# Supplementary material for: Emergence of spatiotemporal invariance in large neuronal ensembles in rat barrel cortex
Source: Front Neural Circuits. 2015 Jul 8;9:34. doi: 10.3389/fncir.2015.00034 (PMC4495341; doi:10.3389/fncir.2015.00034)
Supplement: Supplementary file 1 [file Table1.PDF]

Supplementary Table 1. Onset and peak latencies for each stimulus amplitude. Mean latencies (± s.e.m) for each stimulus amplitude are provided as a reference for grand mean latencies reported throughout the main text. Grand means are repeated at the top of each set of latencies for the sake of comparison, and are the same values as reported in the main text. For s.e.m. calculations for grand means here and in the main text, latencies were first average across stimulus amplitudes for each subject, and then the s.e.m. calculated based on stimulus amplitude means for each subject. Below, statistics are provided for each set of latencies. There was no consistent differences between stimulus amplitudes and most effect sizes, whether significant or not, were small.

For single whisker evoked LFP for the first deflection, onset and peak latencies were not significantly different between stimulus amplitudes (*onset latency,* $F(3,15)=2.81$, $p=0.075$; *peak latency,* $F(3,15)=1.39$, $p=0.284$). For single whisker evoked MUP, significant differences between stimulus amplitudes were found for onset but not peak latencies (*main effects of stimulus amplitude; onset latency,* $F(3,15)=9.60$, $p<0.05$; *peak latency,* $F(3,15)=0.91$, $p=0.460$). For whisker array evoked LFP, onset and peak latencies were not significantly different between stimulus amplitudes (*main effects of stimulus amplitude; onset latency,* $F(3,15)=2.15$, $p=0.13$; *peak latency,* $F(3,15)=1.96$, $p=0.157$). For whisker array evoked MUP, onset and peak latencies were not significantly different between stimulus amplitudes (*main effects of stimulus amplitude; onset latency,* $F(3,15)=3.12$, $p=0.052$; *peak latency,* $F(3,15)=3.09$, $p=0.053$). For repeated single whisker deflections 2-5, peak LFP and MUP latencies were not significantly different between stimulus amplitudes (*LFP,* $F(3,15)=2.87$, $p=0.071$; *MUP,* $F(3,15)=0.35$, $p=0.791$). For repeated whisker array deflections 2-5, there were significant differences between stimulus amplitudes for peak LFP latencies ($F(3,15)=4.32$, $p<0.05$) but not peak MUP latencies ($F(3,15)=1.75$, $p=0.192$).

Onset latencies were shorter for trial averaged MUP than is typically reported for suprathreshold spiking activity in barrel cortex based on spike detection. This is due to differences in the sensitive and nature of each signal. Timestamps of detected spikes are typically aligned with the negative peak of the spike waveform. The negative peak of trial averaged MUP corresponds to overlap in the negative peak of

individual spike waveforms which is more likely closer to the onset of spiking activity. In addition, trial averaged MUP is sensitive to early small amplitude signals in the rising phase of the action potential and possibly to early subthreshold responses with fast temporal components such as synchronized thalamocortical afferents. *All values reported as mean ± s.e.m. 'Mean' refers to the grand mean of subject and stimulus amplitudes.*

Single whisker response latencies

	Onset Latency	Peak Latency				
	Deflection 1 (ms)	*Deflection 1 (ms)*	*Deflection 2 (ms)*	*Deflection 3 (ms)*	*Deflection 4 (ms)*	*Deflection 5 (ms)*
LFP						
**Mean**	**7.6 ± 0.4**	**14.2 ± 0.6**	**19.2 ± 0.9**	**19.7 ± 0.9**	**19.6 ± 0.9**	**19.7 ± 1.0**
0.035°	8.0 ± 0.7	14.4 ± 1.0	20.5 ± 1.4	20.5 ± 1.4	20.3 ± 1.3	20.3 ± 1.5
0.2°	7.9 ± 0.5	14.6 ± 0.7	19.7 ± 1.0	20.1 ± 1.0	20.0 ± 1.0	20.1 ± 1.1
1.25°	7.4 ± 0.3	14.1 ± 0.5	19.5 ± 1.1	20.0 ± 1.1	19.8 ± 1.0	19.9 ± 1.1
7.5°	7.2 ± 0.4	13.6 ± 0.6	17.8 ± 0.6	18.4 ± 0.5	18.4 ± 0.6	18.5 ± 0.7
MUP						
**Mean**	**5.6 ± 0.4**	**8.1 ± 0.5**	**13.6 ± 0.5**	**14.0 ± 0.5**	**14.0 ± 0.5**	**13.7 ± 0.6**
0.035°	5.9 ± 0.4	8.0 ± 0.2	13.6 ± 0.7	13.8 ± 0.9	14.0 ± 0.8	13.8 ± 0.8
0.2°	5.7 ± 0.4	8.2 ± 0.3	13.7 ± 0.6	14.2 ± 0.5	14.0 ± 0.6	13.8 ± 0.6
1.25°	5.6 ± 0.3	7.7 ± 0.2	13.8 ± 0.5	14.4 ± 0.6	14.2 ± 0.5	13.7 ± 0.7
7.5°	5.1 ± 0.4	8.4 ± 0.2	13.3 ± 0.4	13.7 ± 0.4	13.7 ± 0.4	13.5 ± 0.5

Whisker array response latencies

	Onset Latency	Peak Latency				
	Deflection 1 (ms)	*Deflection 1 (ms)*	*Deflection 2 (ms)*	*Deflection 3 (ms)*	*Deflection 4 (ms)*	*Deflection 5 (ms)*
LFP						
**Mean**	**6.0 ± 0.4**	**12.3 ± 0.6**	**18.0 ± 0.7**	**18.2 ± 0.7**	**18.1 ± 0.7**	**17.7 ± 1.0**
0.035°	6.4 ± 0.4	12.9 ± 0.8	17.4 ± 0.9	17.8 ± 0.7	18.0 ± 0.7	17.8 ± 0.8
0.2°	6.1 ± 0.3	12.3 ± 0.7	17.2 ± 0.8	17.9 ± 0.8	17.7 ± 0.9	17.3 ± 1.0
1.25°	5.9 ± 0.3	12.2 ± 0.6	17.5 ± 0.8	18.0 ± 0.9	18.1 ± 0.9	17.5 ± 1.2
7.5°	5.8 ± 0.5	11.9 ± 0.5	20.1 ± 0.7	19.1 ± 0.6	18.8 ± 0.8	18.4 ± 1.2
MUP						
**Mean**	**4.4 ± 0.2**	**6.8 ± 0.4**	**12.2 ± 0.3**	**12.5 ± 0.4**	**12.3 ± 0.4**	**12.1 ± 0.4**
0.035°	4.7 ± 0.3	6.9 ± 0.2	12.1 ± 0.6	12.3 ± 0.5	12.2 ± 0.5	12.1 ± 0.4
0.2°	4.6 ± 0.3	6.9 ± 0.1	11.5 ± 0.6	12.2 ± 0.6	11.9 ± 0.7	11.9 ± 0.6
1.25°	4.2 ± 0.1	6.7 ± 0.1	11.8 ± 0.3	12.4 ± 0.6	12.1 ± 0.6	12.1 ± 0.5
7.5°	4.2 ± 0.1	6.9 ± 0.1	13.2 ± 0.6	13.3 ± 0.4	12.8 ± 0.5	12.4 ± 0.4
