# Supplementary material for: Emergence of spatiotemporal invariance in large neuronal ensembles in rat barrel cortex
Source: Front Neural Circuits. 2015 Jul 8;9:34. doi: 10.3389/fncir.2015.00034 (PMC4495341; doi:10.3389/fncir.2015.00034)

Supplementary Figure 1. Coincident spike waveforms in whisker evoked multi-unit activity. Spontaneous (open arrows) and evoked (closed arrows) multi-unit activity at varying distances from peak responses are plotted for 10 consecutive trials of whisker array stimulation. Note the maximum amplitude of evoked multi-unit potentials which are denoted by dotted red lines in each trace. In (A), far away from peak responses, evoked spike waveforms presumably from individual neurons are comparable in amplitude to spontaneous spikes also presumably from individual neurons. In (B), closer to peak responses, the amplitude of evoked spikes are consistently larger than the amplitude of spontaneous spikes. In (C), the amplitude of evoked spikes are consistently larger than the amplitude of spontaneous spikes *and* the amplitude of evoked spikes at locations further away from peak responses (shown in (A) and (B)). Since spike amplitude should not change if produced by action potentials from a single neuron, these data indicate that these variable spike waveforms are actually produced by multiple coincident spike waveforms with varying numbers of contributing neurons. This greatly constrains the interpretation of traditional spike counts and PSTHs since one “spike” can indicate either a single neuron firing or many neurons firing simultaneously (for a general discussion of this common issue see Bar-Gad, 2001).

Spontaneous and evoked multi-unit activity (300-3k Hz) for 10 consecutive trials

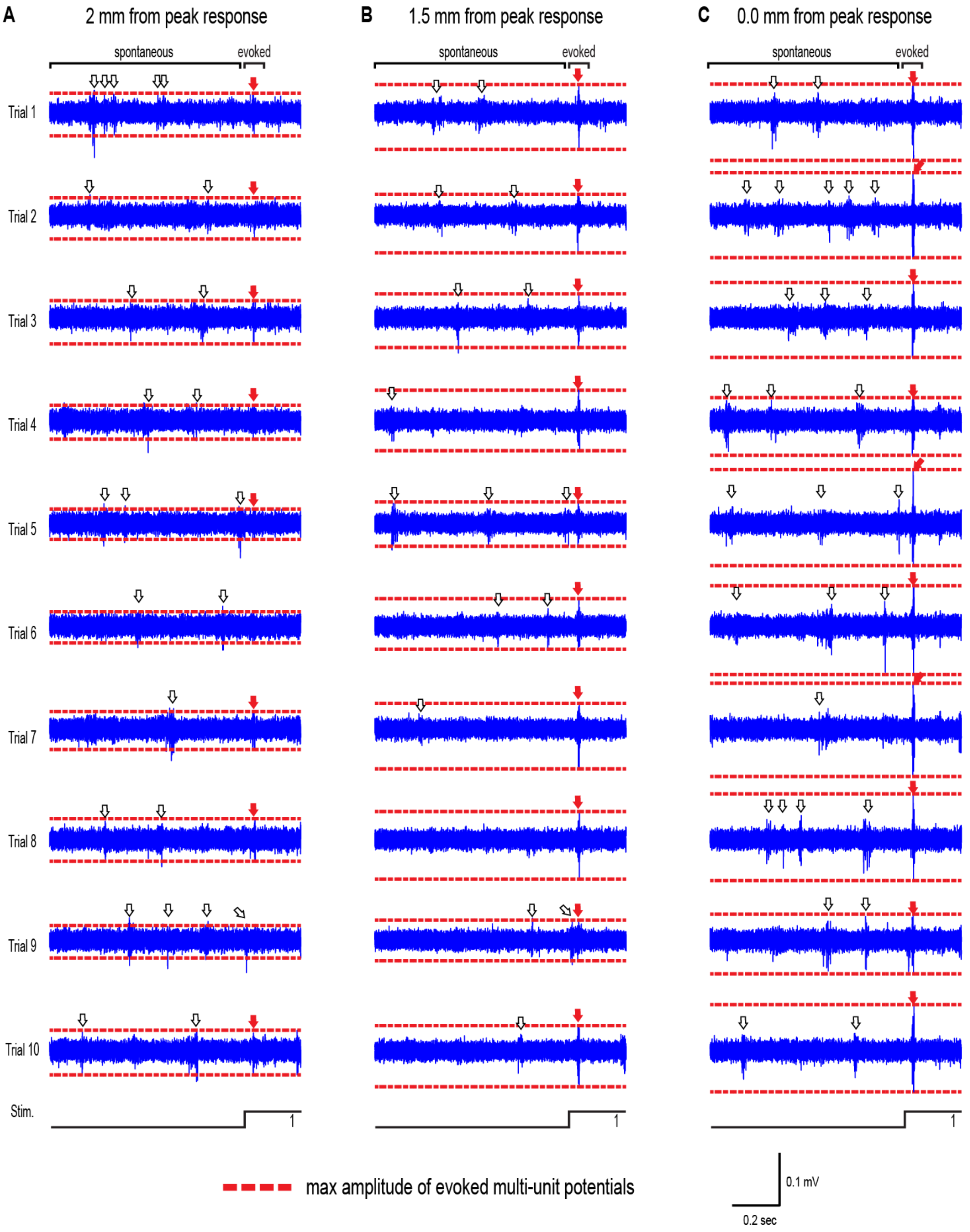

Supplement: Supplementary file 2 [file Image1.PDF]
