# Supplementary material for: Emergence of spatiotemporal invariance in large neuronal ensembles in rat barrel cortex
Source: Front Neural Circuits. 2015 Jul 8;9:34. doi: 10.3389/fncir.2015.00034 (PMC4495341; doi:10.3389/fncir.2015.00034)

Supplementary Figure 2. Multi-unit PSTHs for single whisker (C2) stimuli. (A,B) Raw and normalized movies of averaged (n=6) multi-unit firing rates with 1 ms bin sizes for the first single whisker deflection. Movies in (B) were normalized to the peak firing rate. **(C)** Raw and normalized peak frames of multi-unit firing rates for each of the five single whisker deflections. **(D)** Quantification of magnitude (maximum value; left), spatial profile (PC1, PC2, and PC3 loadings; middle) and similarity between spatial profiles (r^2 value, right) of peak frames for deflections 1-5. *All values are mean ± s.e.m. Compare with **Figure 4** of main text. PSTH results were not preferred due to the presence of overlapping spike waveforms (see **Supp**. **Fig. 1**).*

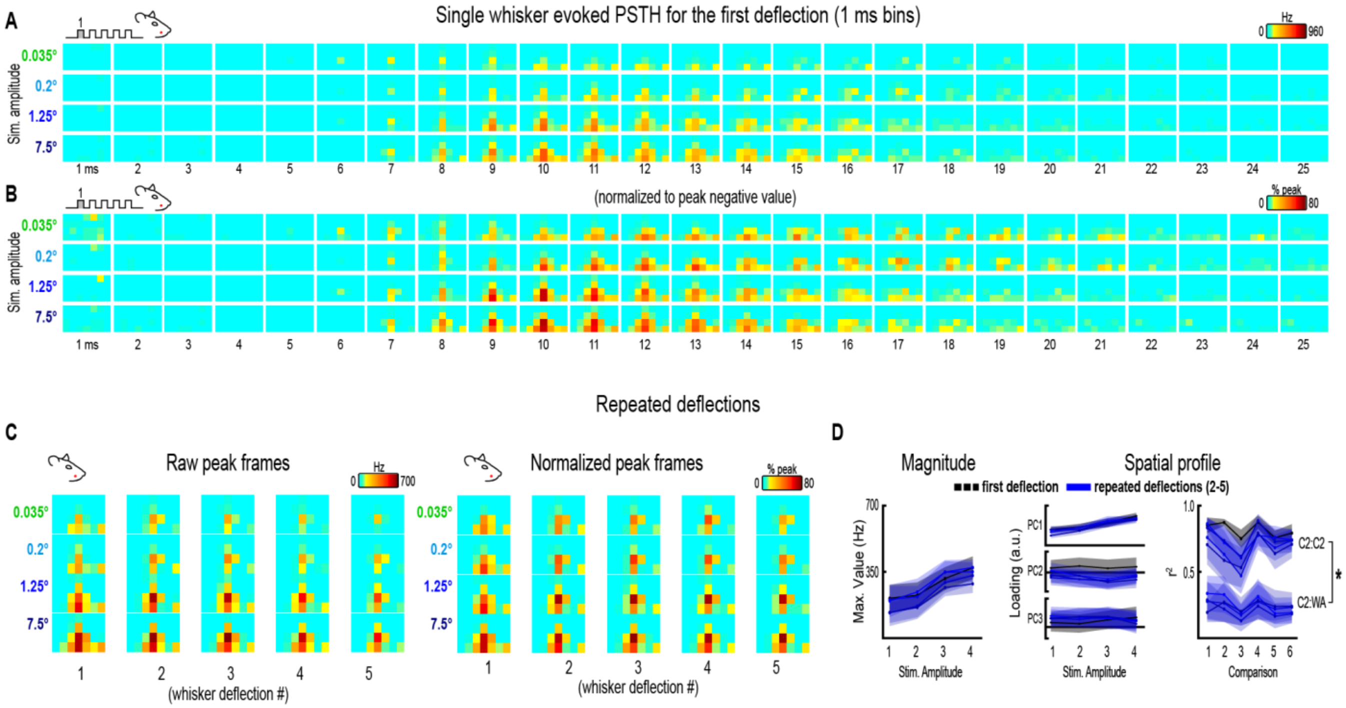

Supplement: Supplementary file 3 [file Image2.PDF]
