# Supplementary material for: Emergence of spatiotemporal invariance in large neuronal ensembles in rat barrel cortex
Source: Front Neural Circuits. 2015 Jul 8;9:34. doi: 10.3389/fncir.2015.00034 (PMC4495341; doi:10.3389/fncir.2015.00034)

Supplementary Figure 3. Multi-unit PSTHs for whisker array stimuli. (A,B) Raw and normalized movies of averaged (n=7) multi-unit firing rates with 1 ms bins for the first single whisker deflection. Movies in (B) were normalized to the peak firing rate in movies for each subject and stimulus amplitude. **(C)** Raw and normalized peak frames of multi-unit firing rates for each of the five single whisker deflections. **(D)** Quantification of magnitude (maximum value; left), spatial profile (PC1, PC2, and PC3 loadings; middle) and similarity between spatial profiles (r^2 value, right) of peak frames for deflections 1-5. Values are mean ± s.e.m. *Compare with **Figure 6** of main text. PSTH results were not preferred due to the presence of overlapping spike waveforms (see **Supp. Fig. 3**).*

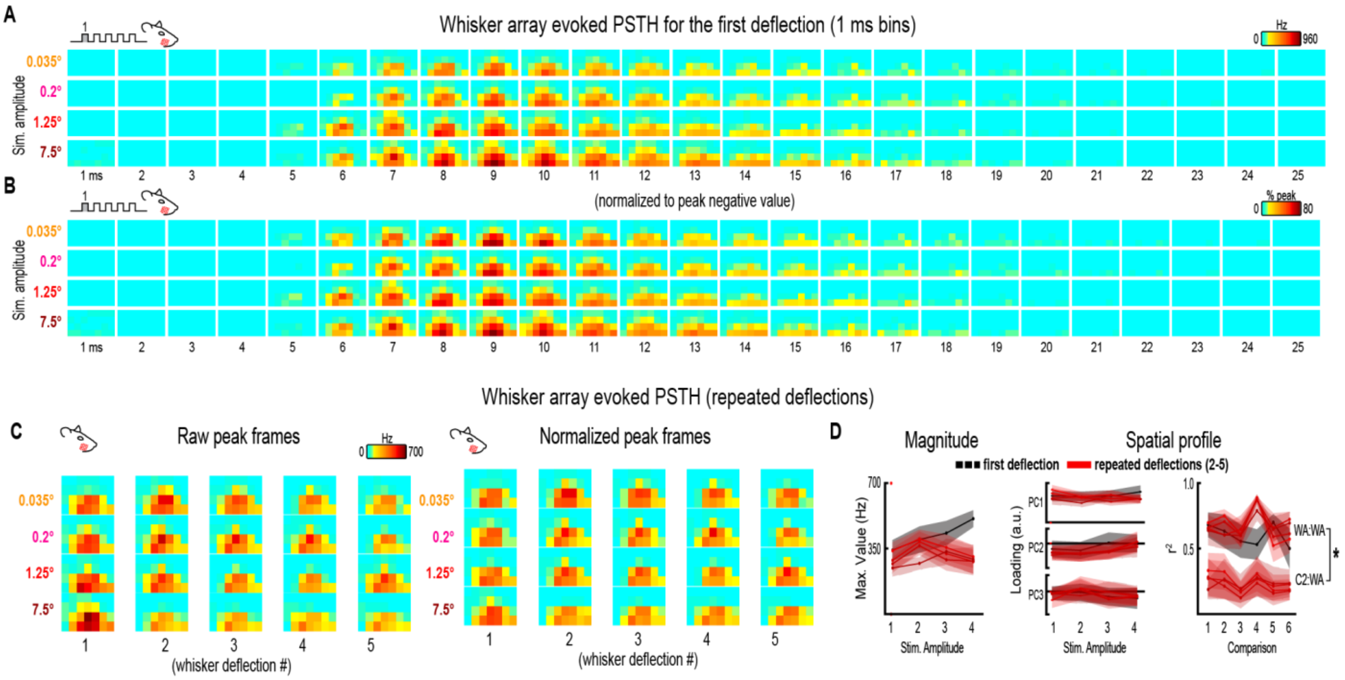

Supplement: Supplementary file 4 [file Image3.PDF]
