# Supplementary material for: Emergence of spatiotemporal invariance in large neuronal ensembles in rat barrel cortex
Source: Front Neural Circuits. 2015 Jul 8;9:34. doi: 10.3389/fncir.2015.00034 (PMC4495341; doi:10.3389/fncir.2015.00034)
Supplement: Supplementary file 5 [file Image4.PDF]

Supplementary Figure 4. Whisker stimulation engages entire mesoscopic field of view for each stimulus amplitude. (A, B) Z-scores were calculated for raw single whisker evoked LFP and MUP at each recording location by dividing by the standard deviation of voltages during the 50 ms period before stimulus onset. Note that evoked LFP and MUP can be detected (> 3 standard deviations above pre-stimulus data, warm colors in images) throughout the entire mesoscopic field of view. **(C, D)** For each recording location, the maximum z-score within the 25 ms of stimulus onset (50 ms for deflections 2-5 since peak latencies tended to be delayed slightly) was calculated. Results for deflection 1, detailed in (A,B), are framed. **(E-H)** Same as (A-D) but for whisker array data.

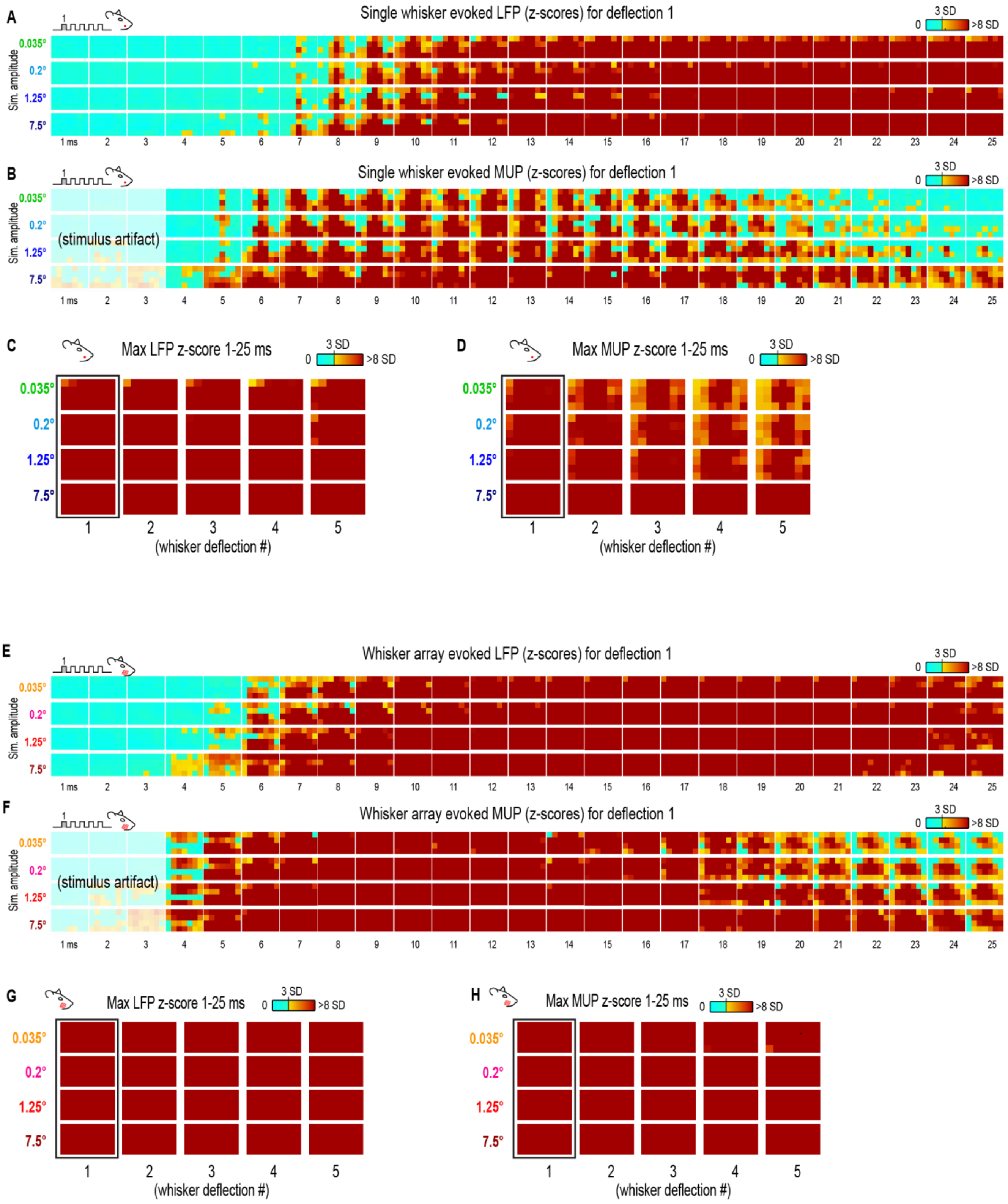

A
Single whisker evoked LFP (z-scores) for deflection 1
3 SD
0
>8 SD
Sim. amplitude
0.035°
0.2°
1.25°
7.5°
1 ms
B
Single whisker evoked MUP (z-scores) for deflection 1
(stimulus artifact)
C
Max LFP z-score 1-25 ms
(whisker deflection #)
D
Max MUP z-score 1-25 ms
(whisker deflection #)
E
Whisker array evoked LFP (z-scores) for deflection 1
F
Whisker array evoked MUP (z-scores) for deflection 1
(stimulus artifact)
G
Max LFP z-score 1-25 ms
(whisker deflection #)
H
Max MUP z-score 1-25 ms
(whisker deflection #)
