# Supplementary material for: Emergence of spatiotemporal invariance in large neuronal ensembles in rat barrel cortex
Source: Front Neural Circuits. 2015 Jul 8;9:34. doi: 10.3389/fncir.2015.00034 (PMC4495341; doi:10.3389/fncir.2015.00034)

Supplementary Figure 5. PCA visualizes differences between single whisker and whisker array evoked activity. PCA was performed on a combined data set with both single whisker and whisker array evoked LFP (A) and MUP (B). Mean PC loadings (n=6), connected in chronological order for each condition, are plotted. In each data set, note that paths for single whisker vs whisker array conditions showed characteristic differences (e.g., in B peanut shaped path only occurs for whisker array conditions). *For each data set, the exact same axes are used for single whisker and whisker array results which were separated for the sake of clarity.*

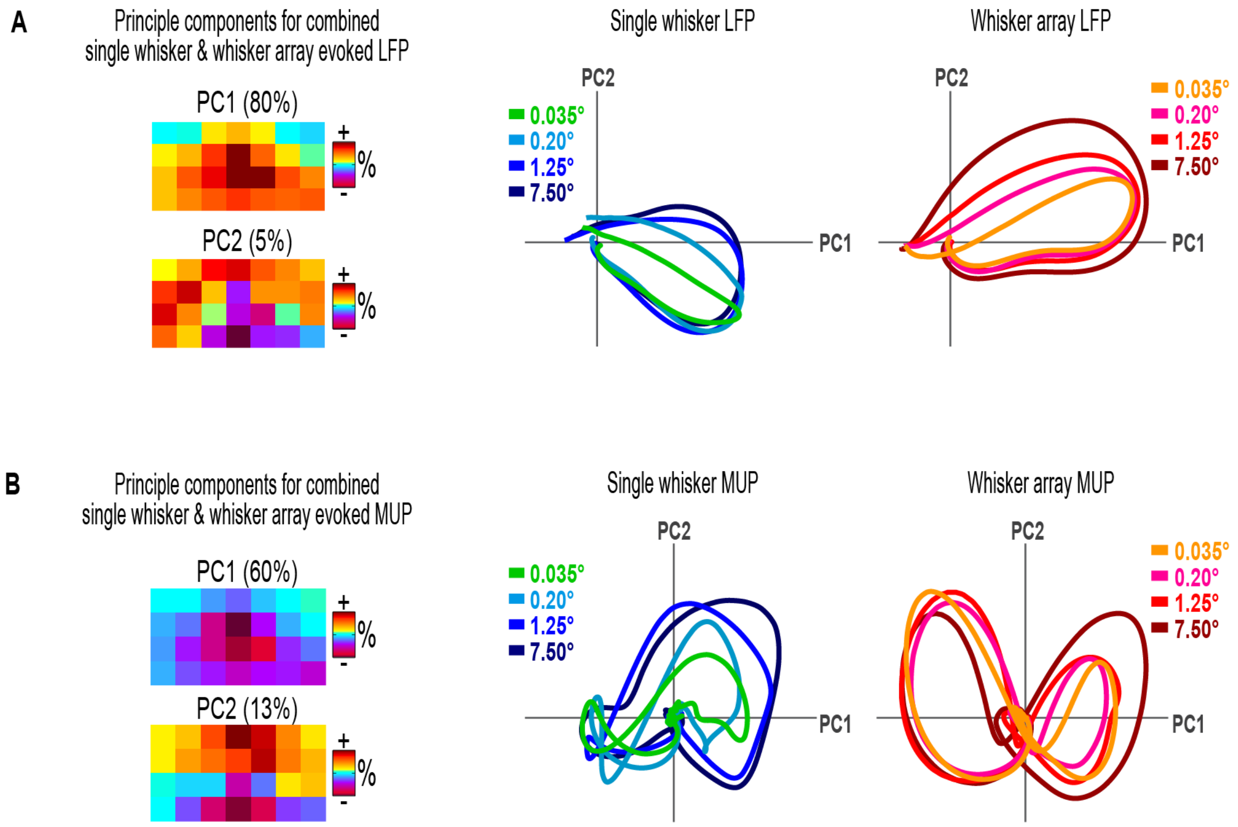

Supplement: Supplementary file 6 [file Image5.PDF]
